# Supplementary material for: The Acute Effects of Schoolbag Loading on Posture and Gait Mechanics in 10- to 13-Year-Old Children: A Cohort from the North West Province
Source: Children (Basel). 2023 Sep 1;10(9):1497. doi: 10.3390/children10091497 (PMC10528080; doi:10.3390/children10091497)
Supplement: Supplementary file 1 [file children-10-01497-s001.zip › Supplementary Table S1.pdf]

Supplementary Table S1: Between-country comparisons for mean differences in relative bag mass.

Comparison	M_{diff}	CI_{upper}	CI_{lower}	p-value
SA-Tunisia	11.61	12.65	10.57	p<0.001
SA-Montenegro	8.65	9.69	7.62	p<0.001
SA-Uganda	8.55	9.66	7.44	p<0.001
SA-Iran	8.54	9.60	7.48	p<0.001
SA-USA	7.61	8.66	6.56	p<0.001
SA-Portugal	5.51	6.64	4.38	p<0.001
SA-India	5.32	6.36	4.27	p<0.001
SA-NZ	5.31	6.56	4.06	p<0.001
SA-Brazil	4.94	5.99	3.89	p<0.001
SA-Ireland	4.51	5.60	3.42	p<0.001
SA-Spain	4.51	5.83	3.19	p<0.001
SA-Ethiopia	4.38	5.47	3.29	p<0.001
SA-Greece	3.44	4.47	2.41	p<0.001
SA-Czech Republic	2.71	3.80	1.62	p<0.001
SA-Netherlands	2.31	3.43	1.19	p<0.001
SA-Italy	0.72	1.77	-0.32	p== 0.694
SA-Poland	0.63	1.73	-0.48	p== 0.791
SA-Malta	0.31	1.36	-0.74	p== 0.791
SA-China	-0.49	0.54	-1.52	p== 0.791
SA-Turkey	-2.99	-1.96	-4.02	p<0.001

Data are shown as mean ± 95%CI; note: SA = South Africa; USA = United States of America; NZ = New Zealand
