# Supplementary material for: The Acute Effects of Schoolbag Loading on Posture and Gait Mechanics in 10- to 13-Year-Old Children: A Cohort from the North West Province
Source: Children (Basel). 2023 Sep 1;10(9):1497. doi: 10.3390/children10091497 (PMC10528080; doi:10.3390/children10091497)
Supplement: Supplementary file 1 [file children-10-01497-s001.zip › Supplementary Table S2.pdf]

Supplementary Table S2: One-way ANOVA results for body mass as a function of gender*age

Comparison	Group1	Group2	Estimate	conf.low	conf.high	p.adj
Gender	F	M	2.39	-3.07	7.85	0.384
Age	10	11	-1.37	-11.31	8.56	0.983
Age	10	12	9.39	-0.39	19.16	0.064
Age	10	13	13.62	3.51	23.73	0.004
Age	11	12	10.76	0.98	20.53	0.026
Age	11	13	14.99	4.88	25.10	0.001
Age	12	13	4.23	-5.72	14.19	0.674
Gender:Age	F:10	M:10	-1.87	-18.62	14.88	1.000
Gender:Age	F:10	F:11	-3.29	-22.24	15.66	0.999
Gender:Age	F:10	M:11	-1.43	-17.38	14.52	1.000
Gender:Age	F:10	F:12	7.75	-12.54	28.03	0.927
Gender:Age	F:10	M:12	9.30	-6.09	24.69	0.552
Gender:Age	F:10	F:13	8.00	-9.30	25.30	0.824
Gender:Age	F:10	M:13	17.09	-0.21	34.38	0.055
Gender:Age	M:10	F:11	-1.42	-19.87	17.03	1.000
Gender:Age	M:10	M:11	0.44	-14.91	15.79	1.000
Gender:Age	M:10	F:12	9.61	-10.21	29.43	0.787
Gender:Age	M:10	M:12	11.16	-3.61	25.93	0.270
Gender:Age	M:10	F:13	9.87	-6.88	26.62	0.584
Gender:Age	M:10	M:13	18.95	2.20	35.70	0.016
Gender:Age	F:11	M:11	1.86	-15.87	19.59	1.000
Gender:Age	F:11	F:12	11.04	-10.67	32.74	0.745
Gender:Age	F:11	M:12	12.59	-4.64	29.81	0.310
Gender:Age	F:11	F:13	11.29	-7.66	30.24	0.570
Gender:Age	F:11	M:13	20.37	1.42	39.32	0.027
Gender:Age	M:11	F:12	9.17	-9.97	28.32	0.797
Gender:Age	M:11	M:12	10.73	-3.13	24.58	0.243
Gender:Age	M:11	F:13	9.43	-6.52	25.38	0.579
Gender:Age	M:11	M:13	18.51	2.57	34.46	0.013
Gender:Age	F:12	M:12	1.55	-17.13	20.23	1.000
Gender:Age	F:12	F:13	0.25	-20.03	20.54	1.000

Gender:Age	F:12	M:13	9.34	-10.94	29.62	0.828
Gender:Age	M:12	F:13	-1.30	-16.69	14.09	1.000
Gender:Age	M:12	M:13	7.79	-7.60	23.18	0.749
Gender:Age	F:13	M:13	9.09	-8.21	26.38	0.713
